# Obscure Injury of Hip

**Published:** 1859-02

**Authors:** W. B. Sprague

**Affiliations:** Westmoreland, N. Y.


ART. V.—*Obscure injury of Hip. Difference of opinion in relation to the Diagnosis. Singular Result.* Reported by W. B. SPRAGUE, M. D., of Westmoreland, N. Y.

DR. FLINT:—I enclose you the following letter, just received from a very intelligent young practitioner. It will serve to illustrate the occasional difficulty of diagnosis in accidents about the hip-joint.

Yours, truly,

F. H. HAMILTON.

*Oct. 9th*, 1856. Mrs. A. B., æt. 24, fell backward from a horse, striking upon the left hip, producing an audible snap, and a severe contusion. Dr. Phelps, of Manchester, was immediately called, and pronounced it a sprain, advising rest, &c. Dr. Loomis, of this place, saw the patient three weeks after, and found the limb shortened from half to three-quarters of an inch, with the leg straight and the foot inverted. He was not satisfied as to the character of the injury. Other surgeons, and among them Dr. Wolcott, of Utica, also examined the case, and were unable to determine the exact nature of the difficulty.

About ten weeks subsequent to the occurrence of the accident, *Sweet*, one of the natural bone-setters, came, and with his usual promptness and *accuracy*, declared every joint on the left side of the body to be dislocated, and accordingly pulled and twisted the patient, hurting her prodigiously, until every joint, from the cranio-vertebral articulation to the toes, passed under his manipulations. He then pronounced her 'all right,' charged twenty-five dollars for his services, abused the 'smart doctors who did n't know what ailed her,' and left a prescription for a liniment, containing seven different sorts, with alcohol and salt; also advising the application every night of a hot pancake to the sole of the foot.

I will not say that she obtained no relief from the preceding treatment.

In November of the following year she went to New Haven, and was there examined by several of the professors in the medical college, whose names she has now forgotten, but who united in pronouncing her case one of fracture of the cervix femoris, recommending her to wear a high-heeled shoe.

Dr. James Sweet, of that city, declared the sciatic nerve to be paralyzed, and prescribed liniment and steaming.

From that time until the night of December 6th, 1858, she suffered much pain and inconvenience, but did nothing in the way of treatment; when, her husband, lying beside her in bed, to relieve a dull, heavy pain in the knee, extended the limb by placing one foot on each side of her ancle and pushing downward, making at the same time counter extension by means of his arms around her chest. Using on this occasion, a more than usual amount of force, (for he had often before operated in the same manner,) the head of the bone seemed to slide, with a snap, into its socket, and she felt immediate relief. Since then, the limb has been rapidly improving in strength and usefulness, and the patient walks easily, and with but a slight halt.

I cannot persuade myself from the above narrative, that this was a dislocation. I think it was a fracture of the neck, but that in the extension made by the husband something has given way which limited the motions of the femur. At some future period this interesting question may perhaps be determined more positively. F. H. H.

